# Whole blood mRNA expression-based targets to discriminate active tuberculosis from latent infection and other pulmonary diseases

**DOI:** 10.1038/s41598-020-78793-2

**Published:** 2020-12-16

**Authors:** Jéssica D. Petrilli, Luana E. Araújo, Luciane Sussuchi da Silva, Ana Carolina Laus, Igor Müller, Rui Manuel Reis, Eduardo Martins Netto, Lee W. Riley, Sérgio Arruda, Adriano Queiroz

**Affiliations:** 1 Advanced Laboratory of Public Health (LASP), Gonçalo Moniz Institute (IGM) / Fiocruz, R. Waldemar Falcão, 121, Candeal, Salvador, BA 40296-710 Brazil; 2 Molecular Oncology Research Center, Barretos Cancer Hospital, R. Antenor Duarte Villela, 1331, Dr. Paulo Prata, Barretos, SP 14784-400 Brazil; 3 Life and Health Sciences Research Institute (ICVS), Medical School, University of Minho, Braga, Portugal; 4 ICVS/3B’s-PT Government Associate Laboratory, Braga, Guimarães, Portugal; 5 Fundação José Silveira, Largo do Campo Santo, s/n-Federação, Salvador, BA 40210-320 Brazil; 6 Division of Infectious Diseases and Vaccinology, School of Public Health, University of California, Berkeley, USA

**Keywords:** Computational biology and bioinformatics, Microbiology, Immunology, Infectious diseases, Diagnostic markers, Predictive markers

## Abstract

Current diagnostic tests for tuberculosis (TB) are not able to predict reactivation disease progression from latent TB infection (LTBI). The main barrier to predicting reactivation disease is the lack of our understanding of host biomarkers associated with progression from latent infection to active disease. Here, we applied an immune-based gene expression profile by NanoString platform to identify whole blood markers that can distinguish active TB from other lung diseases (OPD), and that could be further evaluated as a reactivation TB predictor. Among 23 candidate genes that differentiated patients with active TB from those with OPD, nine genes (CD274, CEACAM1, CR1, FCGR1A/B, IFITM1, IRAK3, LILRA6, MAPK14, PDCD1LG2) demonstrated sensitivity and specificity of 100%. Seven genes (C1QB, C2, CCR2, CCRL2, LILRB4, MAPK14, MSR1) distinguished TB from LTBI with sensitivity and specificity between 82 and 100%. This study identified single gene candidates that distinguished TB from OPD and LTBI with high sensitivity and specificity (both > 82%), which may be further evaluated as diagnostic for disease and as predictive markers for reactivation TB.

## Introduction

Tuberculosis (TB), an aerosol-borne disease caused by *Mycobacterium* *tuberculosis* (Mtb), is one of the top 10 causes of death worldwide and the leading cause of death from a single infectious agent^1^. About a quarter of the world’s population is estimated to have latent TB infection (LTBI), and about 10% of these individuals will progress to have active TB disease during their lifetime (reactivation TB)^1,2^. Despite longstanding intense efforts to control this disease, TB remains a global health problem that mandates better diagnostic tests and preventive strategies.

Worldwide, the diagnosis of active TB is mostly dependent on sputum smear microscopy by Mtb acid-fast staining or culture^3^. Microscopy suffers from low sensitivity, and culture can take several weeks to yield results, and neither can be applied to extrapulmonary TB^4^. Although the GeneXpert MTB/RIF test offers a fast result for active TB, the test can be a challenge for TB diagnosis of children and the elderly due to difficulties in obtaining sputum samples from these groups^5,6^. Also, the GeneXpert MTB/RIF requires sophisticated technology and a well-trained staff, and thus not affordable or sustainable in most healthcare systems^7^. Finally, none of these sputum-based tests can predict reactivation TB.

Multiple populations of immune cells have distinct functions that cooperate for Mtb infection control when the bacillus enters the lungs^8^. During infection, alterations of immune processes in the host lead to changes in the transcriptional profiles of circulating immune cells^9^. The immune response-based biomarker identification for TB diagnosis has extensively been researched^10^ and many of them have focused on distinguishing latent infection from active TB^11–13^. Unfortunately, none of these gene signatures has so far been translated into a point of care (POC) diagnostic test. The translation into the clinical practice of gene signature-based assays is challenged by the difficulty in determining which of the multiple gene signatures can be implemented as a diagnostic platform that is simple and cost-effective.

Here, we report the results of an immune-based gene expression profile study based on the NanoString technology in patients with active TB and other pulmonary diseases (OPD), healthy donors with latent TB infection (LTBI), and uninfected health controls (HC). The aim of this study was to identify whole blood markers that can distinguish active TB from OPD, HC, and LTBI. We identified 23 and seven genes associated with inflammatory mechanisms that distinguished with high sensitivity and specificity, patients with TB from OPD and LTBI, respectively.

## Results

### Demographic and clinical characteristics of the study population

The demographic, clinical, and laboratory features of the 35 study participants are shown in Table 1. Of the 17 TB patients, 13 (76.5%) had sputum smear test positive, three were positive by Mtb culture and one patient had the TB confirmed by Mtb molecular test (XPERT TB/RIF). Of all TB patients, eight (47.1%) were screened by Mtb culture. The median age was 41.9 (± 14.04) years in the TB group, 42.7 (± 17.06) in the LTBI group, 43.8 (± 9.70) in the OPD group, and 32.5 (± 3.53) in the HC group.

**Table 1:** Demographic and clinical data of study population.

	Active TB 17	LTBI 7	OPD 5	HC 6
**Gender, n (%)**^a^				
Men	11 (68.75)	4 (57.14)	2 (40)	1 (20)
Women	5 (31.25)	3 (42.86)	3 (60)	4 (80)
Age ± SD^c^	41.93 ± 14.04	42.67 ± 17.06	43.8 ± 9.70	32.5 ± 3.53
**TB diagnosis, n (%)**				
Sputum Smear				
Pos	13 (76.5)	–	0	
Neg	3 (23.5)	–	5 (100)	–
Culture				
Pos	8 (47.1)	–	0	–
Neg	0	–	5 (100)	–
N/S	9 (52.9)	–	–	–
Xpert MTB/RIF	1 (5.9)	–	–	–
**IGRA: n (%)**				
Pos	–	7 (100)	–	0
Neg	–	0	–	6 (100)

*TB* tuberculosis, *LTBI* latent tuberculosis infection, *OPD* other pulmonary disease, *HC* health communicants, *SD* standard deviation, *N/S* not screened.

^a^Missing data: gender (TB = 1 and HC = 1) and age (TB = 1, TBL = 1 and HC = 1).

### Sample clustering

**Figure 1:**
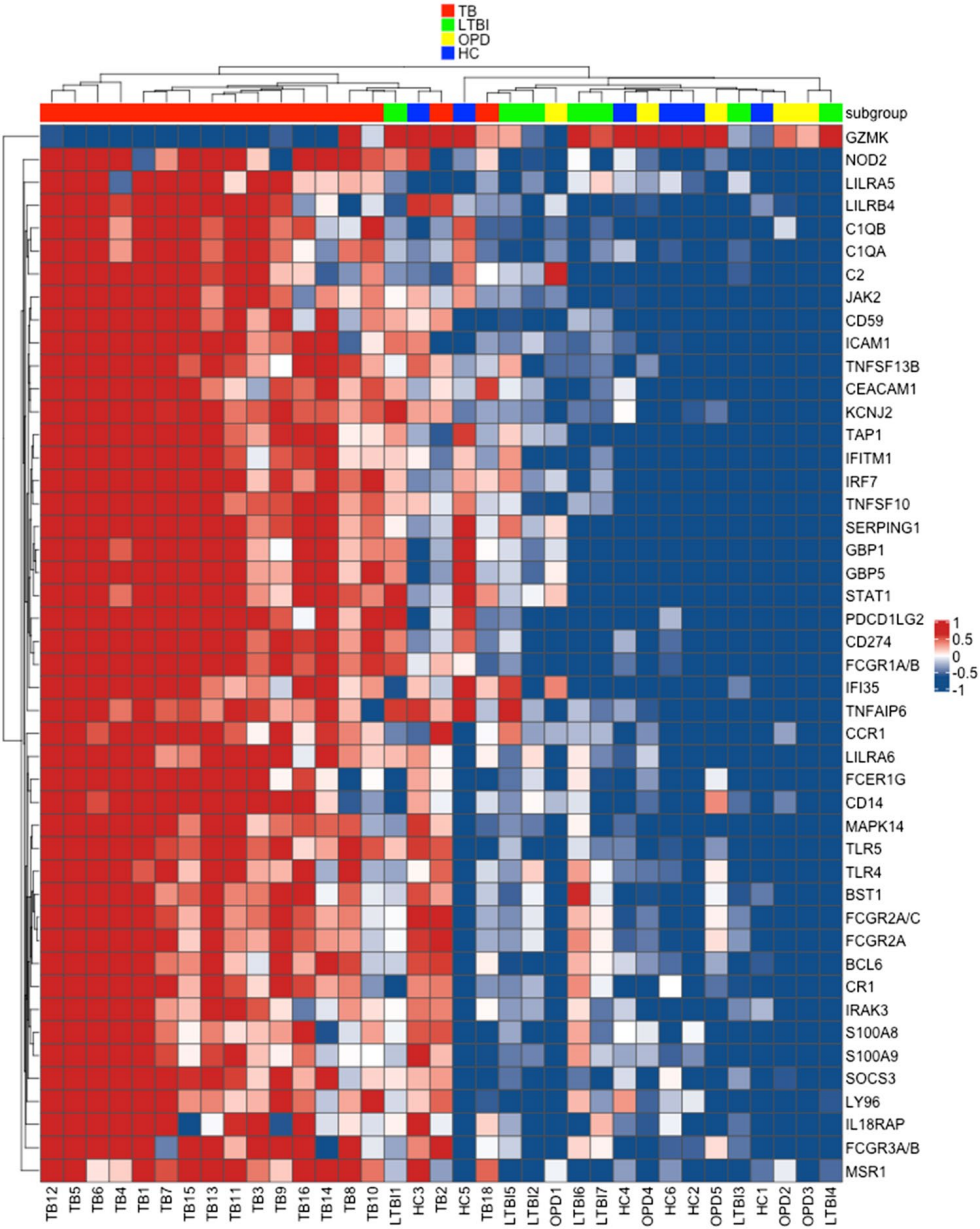
Heatmap showing different expression pattern of 46 proinflammatory genes out of 549 genes. Heatmap of gene expression levels in patients diagnosed with active TB (red), LTBI subjects (green), OPD patients (yellow) and uninfected donors HC (blue). Expression levels are scaled from dark blue (low expression) to dark red (high expression). Heatmap was generated in R (version 3.6.3) with the ComplexHeatmap package (version 2.0.0, https://bioconductor.org/packages/release/bioc/html/ComplexHeatmap.html).

We evaluated 594 inflammatory genes in whole blood from 17 TB patients and 18 controls (seven with LTBI, six HC and five with OPD). We further organized these groups in order to identify whole blood biomarkers to diagnose active TB (TB vs. OPD) and candidate to predict TB reactivation (LTBI vs. TB). First, we evaluated all four study groups together to verify whether the gene panel would be able to distinguish them. Figure 1 shows a heatmap of the normalized data generated via unsupervised hierarchical clustering. The mRNA expression levels of 46 of 594 genes segregated the study groups into two large groups. Transcripts that showed increased expression (red) clustered among TB patients while those that showed decreased expression clustered among non-active TB groups. Two individuals belonged to the groups LTBI (LTBI1) and HC (HC3) clustered with patients with active TB.

### Gene expression data of TB and OPD donors

**Figure 2:**
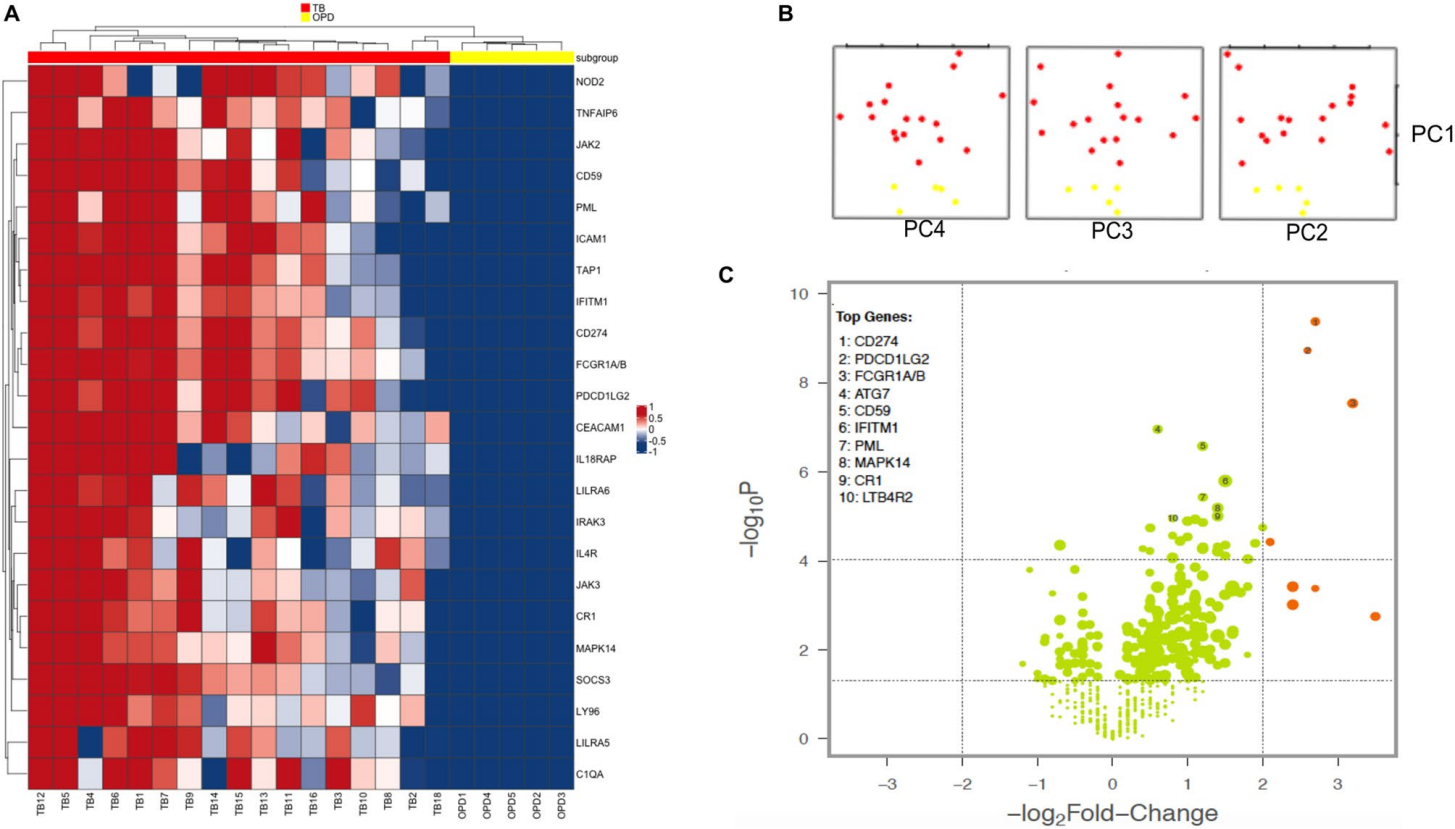
Identification of markers for TB diagnosis. (**A**) Heatmap of 23 gene expression levels of TB (red) and OPD (yellow) patients. (**B**) PCA score plot of TB and OPD patients. (**C**) Volcano plots showing the distribution of the gene expression fold changes in TB patients relative to OPD patients. Genes with absolute fold change ≥ 4 and p-value ≤ 0.05 are indicated in orange. Expression levels are scaled from dark blue (low expression) to dark red (high expression). Figures were generated with R (version 3.6.3) using *ComplexHeatmap* package (version 2.0.0, https://bioconductor.org/packages/release/bioc/html/ComplexHeatmap.html), *prcomp* function from *stats* package (version 3.6.3, https://www.r-project.org/), and NanoStringNorm package (version 1.2.1.1, https://cran.r-project.org/web/packages/NanoStringNorm/index.html) for heatmap, pca and Volcano plot, respectively.

Asthma represents a chronic non-infectious inflammatory airways disease and needs to be promptly distinguished from TB by healthcare providers. We identified 23 candidates genes that differentiated most of the TB patients from asthma (OPD group) (p < 0.001 and fold change [FC] > 2) (Fig. 2A). Principal component analyses (PCA) of the gene expression data showed significant separation between TB and OPD patients (Fig. 2B). The findings are also presented by the volcano plots of all data displayed in orange at a significance level of p < 0.05 and at a log2-fold change higher than 2 for both groups (Fig. 2C). These analyses identify genes that can be used to distinguish TB and OPD patients, which included *CD274*, *PDCD1LG2* and *FCGR1A/B* (p-value < 0.0001 and log2-fold change ratio > 2.6) (Fig. 2C).

### Gene expression data of TB and LTBI donors

**Figure 3:**
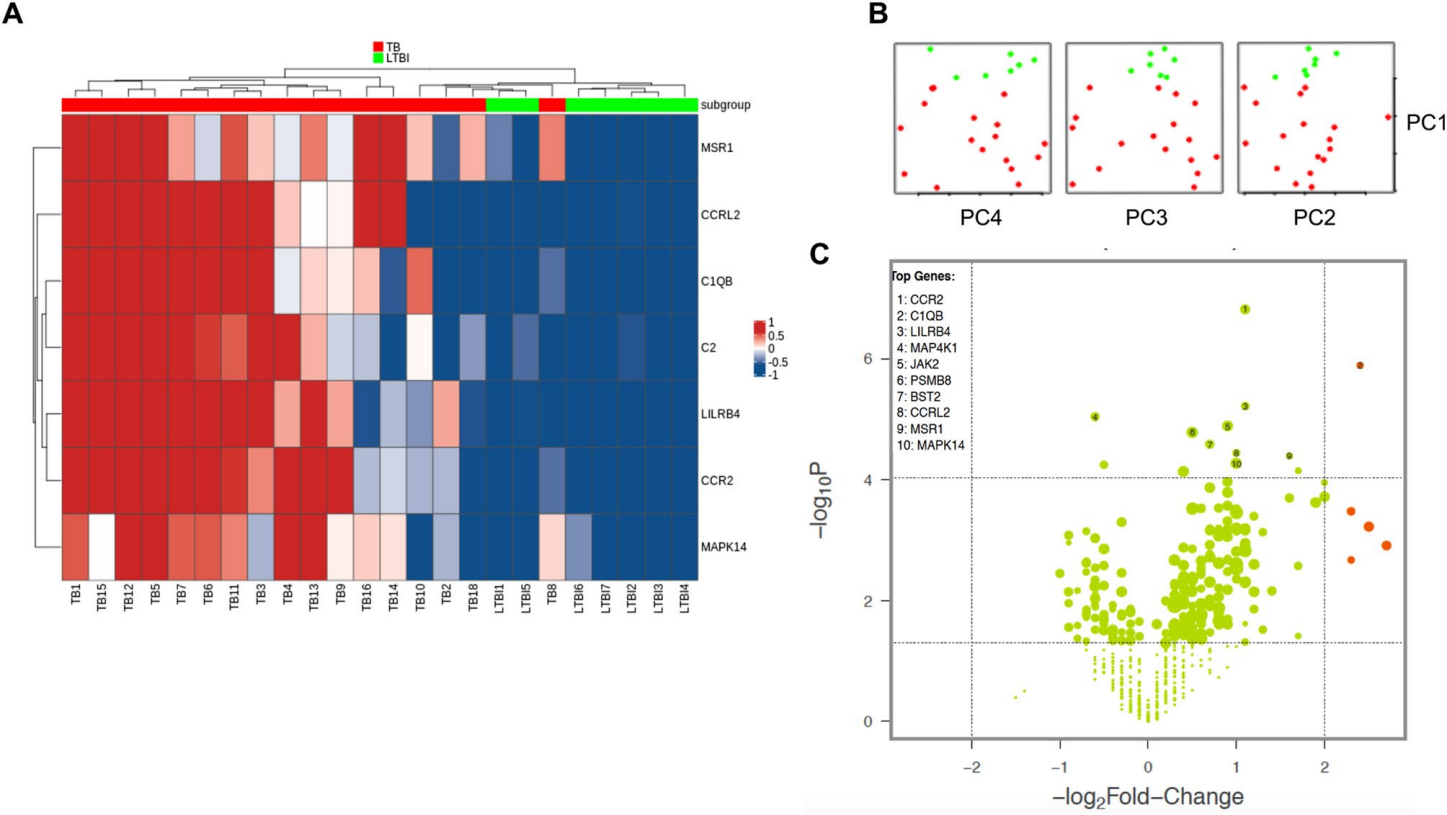
Identification of markers for TB progression. (**A**) Heatmap of seven gene expression levels of TB (red) and LTBI (green) subjects. (**B**) PCA score plot of TB and LTBI subjects. (**C**) Volcano plot showing the distribution of the gene expression fold changes in TB patients relative to LTBI. Genes with absolute fold change ≥ 4 and p-value ≤ 0.05 are indicated in orange. Expression levels are scaled from dark blue (low expression) to dark red (high expression). Figures were generated with R (version 3.6.3) using *ComplexHeatmap* package (version 2.0.0, https://bioconductor.org/packages/release/bioc/html/ComplexHeatmap.html), *prcomp* function from *stats* package (version 3.6.3, https://www.r-project.org/), and NanoStringNorm package (version 1.2.1.1, https://cran.r-project.org/web/packages/NanoStringNorm/index.html) for heatmap, pca and Volcano plot, respectively.

We also compared the gene expression levels between TB and LTBI groups aiming to identify candidate markers able to differentiate these groups. Both heatmap (Fig. 3A) and PCA analysis (Fig. 3B) show 7 of 594 inflammatory genes that significantly differentiate those groups (p < 0.001 and FC > 2). Volcano plots analyses revealed two promising genes (*CCR2* and *CIQB*, p-value < 0.0001 and log2-fold change ratio > 1.1 and 2.4, respectively) that can be further tested as a possible marker of TB reactivation (Fig. 3C).

### Receiver operating characteristic (ROC) curve analysis

ROC analysis was used to evaluate the individual discriminatory performance of the genes that showed a p-value < 0.001 on the heatmap for the study group’s comparison. The values of area under the curve (AUC), sensitivity, specificity, and the optimal cut-off points for TB diagnostic tests (Table 2) and to differentiate TB and LTBI subjects (Table 3) are shown. *CD274,* *CEACAM1,* *CR1,* *FCGR1A/B,* *IFITM1,* *IRAK3,* *LILRA6,* *MAPK14,* *PDCD1LG2* genes (all of them presented AUC = 1.0, 100% of sensitivity and specificity) seems promising targets to distinguish TB and OPD patients (Table 2) (see Supplementary Fig. S1). Table 3 presents seven possible candidates to be further evaluated as a predictor of TB progression, including the CCR2, which showed an AUC = 1.0 and both sensitivity and specificity of 100% (see Supplementary Fig. S2).

**Table 2:** ROC analysis, sensibility and specificity of candidate genes to TB diagnosis.

Gene	ROC AUC (95% CI)	ROC p-value	Sensitivity % (95% CI)	Specificity % (95% CI)	Cut-off
C1QA	0.98 (0.95–1.02)	< 0.0001	88.24 (63.56–98.54)	100 (66.37–100)	> 3.74
CD274	1.00	< 0.0001	100 (80.49–100)	100 (66.37–100)	> 5.71
CD59	0.98 (0.95–1.02)	< 0.0001	94.12 (71.31–99.85)	100 (66.37–100)	> 8.63
CEACAM1	1.00	< 0.0001	100 (80.49–100)	100 (66.37–100)	> 6.02
CR1	1.00	< 0.0001	100 (80.49–100)	100 (66.37–100)	> 10.02
FCGR1A/B	1.00	< 0.0001	100 (80.49–100)	100 (66.37–100)	> 7.83
ICAM1	0.98 (0.95–1.02)	< 0.0001	88.24 (63.56–98.54)	100 (66.37–100)	> 7.54
IFITM1	1.00	< 0.0001	100 (80.49–100)	100 (66.37–100)	> 12.86
IL18RAP	0.98 (0.95–1.02)	< 0.0001	88.24 (63.56–98.54)	100 (66.37–100)	> 8.97
IL4R	0.98 (0.95–1.02)	< 0.0001	94.12 (71.31–99.85)	100 (66.37–100)	> 9.08
IRAK3	1.00	< 0.0001	100 (80.49–100)	100 (66.37–100)	> 6.86
JAK2	0.99 (0.97–1.01)	< 0.0001	94.12 (71.31–99.85)	100 (66.37–100)	> 9.86
JAK3	0.99 (0.97–1.01)	< 0.0001	94.12 (71.31–99.85)	100 (66.37–100)	> 8.59
LILRA5	0.97 (0.92–1.02)	< 0.0001	88.24 (63.56–98.54)	100 (66.37–100)	> 9.87
LILRA6	1.00	< 0.0001	100 (80.49–100)	100 (66.37–100)	> 7.79
LY96	0.99 (0.97–1.01)	< 0.0001	94.12 (71.31–99.85)	100 (66.37–100)	> 7.69
MAPK14	1.00	0.0008757	100 (80.49–100)	100 (47.82–100)	> 10.03
NOD2	0.92 (0.81–1.04)	0.004259	82.35 (56.57–96.20)	100 (47.82–100)	> 8.79
PDCD1LG2	1.00	0.0008757	100 (80.49–100)	100 (47.82–100)	> 4.13
PML	0.98 (0.95–1.02)	0.001156	94.12 (71.31–99.85)	100 (47.82–100)	> 7.72
SOCS3	0.96 (0.88–1.04)	0.001981	94.12 (71.31–99.85)	100 (47.82–100)	> 7.40
TAP1	0.97 (0.92–1.03)	0.001518	88.24 (63.56–98.54)	100 (47.82–100)	> 8.63
TNFAIP6	0.98 (0.95–1.02)	0.001156	94.12 (71.31–99.85)	100 (47.82–100)	> 6.02

**Table 3:** ROC analysis, sensibility and specificity of candidate genes to predict TB reactivation.

Gene	ROC AUC (95% CI)	ROC p-value	Sensitivity % (95% CI)	Specificity % (95% CI)	Cut-off
C1QB	0.97 (0.92–1.02)	0.0003360	88.24 (63.56–98.54)	100 (59.04–100)	> 4.15
C2	0.92 (0.82–1.02)	0.001348	82.35(56.57–100)	100 (59.04–100)	> 4.16
CCR2	1.00	0.0001594	100 (80.49–100)	100 (59.04–100)	> 8.61
CCRL2	0.86 (0.71–1.01)	0.005753	82.35 (56.57–96.20)	85.71 (42.13–99.64)	> 5.31
LILRB4	0.94 (0.82–1.05)	0.0008613	94.12 (71.31–99.85)	100 (59.04–100)	> 6.56
MAPK14	0.95 (0.88–1.03)	0.0005420	88.24 (63.56–98.54)	100 (59.04–100)	> 10.54
MSR1	0.99 (0.96–1.01)	0.0002051	94.12 (71.31–99.85)	100 (59.04–100)	> 4.72

## Discussion

The World Health Organization (WHO) identified the need for a non-sputum-based test as a high-priority for TB diagnosis and suggested that a rapid biomarker-based test should be easy to perform and implement at health posts; should increase the number of patients diagnosed with TB; should have sensitivity > 98% among patients with smear-positive, culture-positive, and ≥ 68% for smear-negative and culture-positive pulmonary TB in adults; and the test would ideally be able to diagnose adults and children, and pulmonary TB and extrapulmonary TB alike^14^. Here, we performed a multiplex gene expression analysis in a single assay for more than 500 inflammatory genes in whole blood samples. By this approach, of all 30 genes herein identified, 23 were candidate targets to diagnose active TB and seven can be validated as biomarkers to distinguish LTBI and TB. All those 30 genes showed sensitivity and specificity > 82%, and ROC AUC > 0.8.

A major challenge to interrupt the TB transmission cycle is to predict when an individual with LTBI will develop active TB. Here, we identified seven genes that were able to discriminate TB patients from LTBI individuals, all presenting high sensitivity and specificity in ROC curve analysis (Table 3). The expression of five (*CCRL2,* *C1QB,* *C2,* *LILRB4,* *and* *CCR2*) of seven genes placed the donor TB8 (TB patient) in the cluster enriched by the LTBI group (Fig. 3). It is possible that the other two genes (*MSR1* and *MAPK14*), which shared a pattern of expression similar to the TB patients, maybe the first set of genes to undergo a change in the level of expression during progression to active TB. To confirm these findings, it is necessary to carry out an evaluation of the expression of these genes in a cohort with LTBI subjects.

We identified 30 candidate genes to be further tested for TB diagnosis and as biomarkers for TB progression. From 23 genes suggested to be suitable for TB diagnosis, ten were related to adaptive immune response, ten were involved in innate immune response, and the other three genes (*JAK2*, *JAK3,* and *LY96*) were not specifically related to either. Conversely, for TB progression, five of seven genes were components of the innate immune system and were increased in TB patients relative to LTBI volunteers (Table 4). These data suggest the involvement of activation of the innate immune response during progression to active TB in latently infected subjects.

**Table 4:** Annotation of selected genes based on ROC curve analysis.

Symbol	Name	Annotation	Target for
C1QA	Complement C1q A chain	Complement system, host pathogen interaction, innate immune system	TB diagnosis
C1QB	Complement C1q B chain	Complement system, host pathogen interaction, innate immune system	TB progression
C2	Complement component 2	Complement system, host pathogen interaction, innate immune system	TB progression
CCR2	C–C chemokine receptor type 2	Chemokine signaling, cytokine signaling, innate immune system, lymphocyte activation	TB progression
CCRL2	C–C chemokine ligand type 2	Chemokine signaling	TB progression
CD59	CD59 molecule	Complement system, innate immune system, lymphocyte activation	TB diagnosis
CD274	CD274 molecule	Adaptive immune system, cell adhesion, lymphocyte activation	TB diagnosis
CEACAM1	CEA cell adhesion molecule 1	Hemostasis, innate immune system and lymphocyte activation	TB diagnosis
CR1	Complement receptor type 1	Complement system, host pathogen interaction, innate immune system	TB diagnosis
FCGR1A/B	Fc fragment of IgG receptor Ia	Adaptive immune system, cytokine signaling, host pathogen interaction, innate immune system, MHC class I antigen presentation, phagocytosis and degradation and Type II interferon signaling	TB diagnosis
ICAM1	Intercellular adhesion molecule 1	Adaptive immune system, cell adhesion, innate immune system, lymphocyte activation	TB diagnosis
IFITM1	Interferon induced transmembrane protein 1	Adaptive immune system, B cell receptor signaling, cytokine signaling and Type I interferon signaling	TB diagnosis
IL18RAP	Interleukin 18 receptor accessory protein	Cytokine signaling, oxidative stress	TB diagnosis
IL4R	Interleukin 4 receptor	Cytokine signaling, lymphocyte activation and Th2 differentiation	TB diagnosis
IRAK3	Interleukin 1 receptor associated kinase 3	Cytokine signaling, innate immune system and TLR signaling	TB diagnosis
JAK2	Janus kinase 2	Chemokine signaling, cytokine signaling, host pathogen interaction, hemostasis, oxidative stress, Th1 and Th17 differentiation, Type II interferon signaling	TB diagnosis
JAK3	Janus kinase 3	Chemokine signaling, cytokine signaling, host pathogen interaction, hemostasis, lymphocyte activation, Th2 and Th17 differentiation	TB diagnosis
LILRA5	Leukocyte immunoglobulin like receptor A5	Adaptive immune system	TB diagnosis
LILRA6	Leukocyte immunoglobulin like receptor A6	Adaptive immune system and MHC class I antigen presentation	TB diagnosis
LILRB4	Leukocyte immunoglobulin like receptor B4	Adaptive immune system	TB progression
LY96	Lymphocyte antigen 96	Adaptive immune system, apoptosis, host pathogen interaction, innate immune system, MHC class I antigen presentation, NF-κB signaling, TLR signaling	TB diagnosis
MAPK14	Mitogen-activated protein kinase 14	Cytokine signaling, hemostasis, host pathogen interaction, innate immune system, lymphocyte trafficking, NLR signaling, T cell receptor signaling, Th17 differentiation, TNF family signaling and TLR signaling	TB diagnosis and TB progression
NOD 2	Nucleotide binding oligomerization domain containing 2	Cytokine signaling, host pathogen interaction, innate immune system, lymphocyte activation, NLR signaling, TNF family signaling, TLR signaling	TB diagnosis
MSR1	Macrophage scavenger receptor 1	Phagocytosis and degradation	TB progression
PDCD1LG2	Programmed cell death 1 ligand 2	Adaptive immune system, cell adhesion and lymphocyte activation	TB diagnosis
PML	Promyelocytic leukemia	Cytokine signaling, host pathogen interaction, oxidative stress, Type II interferon signaling	TB diagnosis
SOCS3	Suppressor of cytokine signaling 3	Adaptive immune system, cytokine signaling, host pathogen interaction, MHC class I antigen presentation, TNF family signaling, Type I interferon signaling and Type II interferon signaling	TB diagnosis
TAP1	Transporter 1, ATP binding cassette subfamily B member	Adaptive immune system, host pathogen interaction, MHC class I antigen presentation, phagocytosis and degradation	TB diagnosis
TNFAIP6	Tumor necrosis factor alpha induced protein 6	Innate immune system	TB diagnosis

*TB* Tuberculosis, *LTBI* latent tuberculosis infection.

Previously identified genes that can discriminate TB patient from non-TB patients and TB risk^11,13,15–25^ either do not fill the minimum sensitivity requirements in adults regardless of HIV status for a POC test (95% in smear-positive culture-confirmed cases and 60–80% in smear-negative culture-confirmed cases), or they proposed gene signatures-based tests which are very difficult to implement. Here, although the number of participants was a limiting issue, we identified single candidate genes for TB diagnosis and progression, all of them presenting high levels of AUC, sensitivity, and specificity.

This study provided valuable information on the development of new diagnostic tests for TB. When validated in a larger population-based study, the expression of the genes herein identified can compose new tools that will overcome the limitations of the currently available diagnostic tests, including low sensibility, long time consuming to perform, and requirement of sputum samples collection. Besides, some of the genes can distinguish seek people with TB from those latently infected. These targets need to be further validated as a possible biomarker to predict TB reactivation in a prospective cohort study.

## Methods

### Study participants

Subjects were recruited between November 2015 to December 2016. Written informed consent was obtained from all participants. Our study included 35 participants, 17 active TB, and 18 controls from which seven were healthy donors with latent *M.* *tuberculosis* infection (LTBI), six were uninfected health controls (HC), and five were patients with asthma (OPD). All participants were recruited at the Instituto Brasileiro para Investigação de Tuberculose (IBIT), Bahia, Brazil and 2° Centro de Saúde Rodrigo Argolo, Bahia, Brazil. TB patients were confirmed to have active pulmonary TB by chest X-ray and at least sputum smear microscopy and/or culture positive. Symptomatic patients with sputum smear microscopy negative had TB confirmed by TB culture. TB patients with no sputum smear microscopy and/or culture screened had TB diagnosis by the Xpert MTB-Rif system. The blood sample was collected prior to TB treatment. Household contacts of TB patients were defined as belonging to either LTBI or HC groups, according to QuantiFERON-TB (QFT) Gold In-Tube test. Those with QTF Gold In-tube test negative (cut-off ≤ 0.35 IU/mL) were considered healthy controls while the household contacts with positive results (cut-off > 0.35 IU/mL) were considered LTBI patients. OPD group was composed of patients who sought care with suspected pulmonary TB but were negative to both sputum smear microscopy and culture. Individuals who tested positive for human immunodeficiency virus and patients taking immunosuppressive drugs were excluded. All subjects were between 18 and 65 years old.

### RNA isolation

For each donor, we collected 2.5 mL peripheral blood in a PAXgene blood RNA tube (PreAnalytiX). RNA was isolated and purified with the PAXgene Blood RNA kit (Qiagen), according to the manufacturer’s protocol for gene expression analysis by NanoString technology. RNA quantification and quality were assessed by Nanodrop.

### NanoString

We performed gene expression assays at the Molecular Oncology Research Center, Barretos Cancer Hospital, Barretos, Brazil using the NanoString technology with nCounter Immunology Panel that contains 594 targets and 15 internal reference genes. Up to 100 ng of total RNA per sample was used and protocol was performed according to manufacturer’s recommendations. Briefly, RNA was hybridized with reporter and capture probes (NanoString Technologies) and incubated at 67 °C for 21 h. Samples were then loaded onto automated nCounter Prep Station (NanoString, Technologies) for sample purification and immobilization in cartridges. Finally, cartridges were transferred to nCounter Digital Analyzer (NanoString Technologies) to capture image in 280 fields of view (FOVs) providing all gene counts.

### Data analysis

The files corresponding to each cartridge were initially analyzed in nSolver Software (NanoString Technologies) for quality control assessment. Then, we analyzed the data in R statistical environment (version 3.6.3)^26^. Distributions of raw counts were evaluated in quantro package^27^. Normalization and differential expression were carried out with NanoStringNorm package^28^. Raw data were normalized with the geometric mean of positive control and housekeeping genes. Hierarchical clustering with Pearson correlation coefficient distance of differentially expressed genes was performed on ComplexHeatmap package^29^. The ability of genes to discriminate the study groups was evaluated with receiver operating characteristic (ROC) curves and the graphic representation was created by the statistical analysis system GraphPad Prism.

### Ethical statement

The study was approved by the Research Ethics Council (CEP) of Maternidade Climério de Oliveira from Universidade Federal da Bahia, CAAE: 48844315.8.0000.5543. Following the basic norms of CEP, Resolution 466/12, all study participants were verbally and in writing informed about the objectives of the study, their participation and IRB contacts and the study coordinator. All participants signed the Consent Form, assuring confidentiality and liberty to leave the study and all methods were performed in accordance with the relevant guidelines and regulations.

## Supplementary Information

Supplementary Information.
