# Supplementary material for: Whole blood mRNA expression-based targets to discriminate active tuberculosis from latent infection and other pulmonary diseases
Source: Sci Rep. 2020 Dec 16;10:22072. doi: 10.1038/s41598-020-78793-2 (PMC7745039; doi:10.1038/s41598-020-78793-2)
Supplement: Supplementary file 1 — Supplementary Information. [file 41598_2020_78793_MOESM1_ESM.pdf]

# Supplementary information

## Whole blood mRNA expression-based targets to discriminate active tuberculosis from latent infection and other pulmonary diseases

Jéssica D. Petrilli, Luana E. Araújo, Luciane Sussuchi, Ana Carolina Laus, Igor Müller, Rui Manuel Reis, Eduardo Martins Netto, Lee W. Riley, Sérgio Arruda, Adriano Queiroz.

## Figures

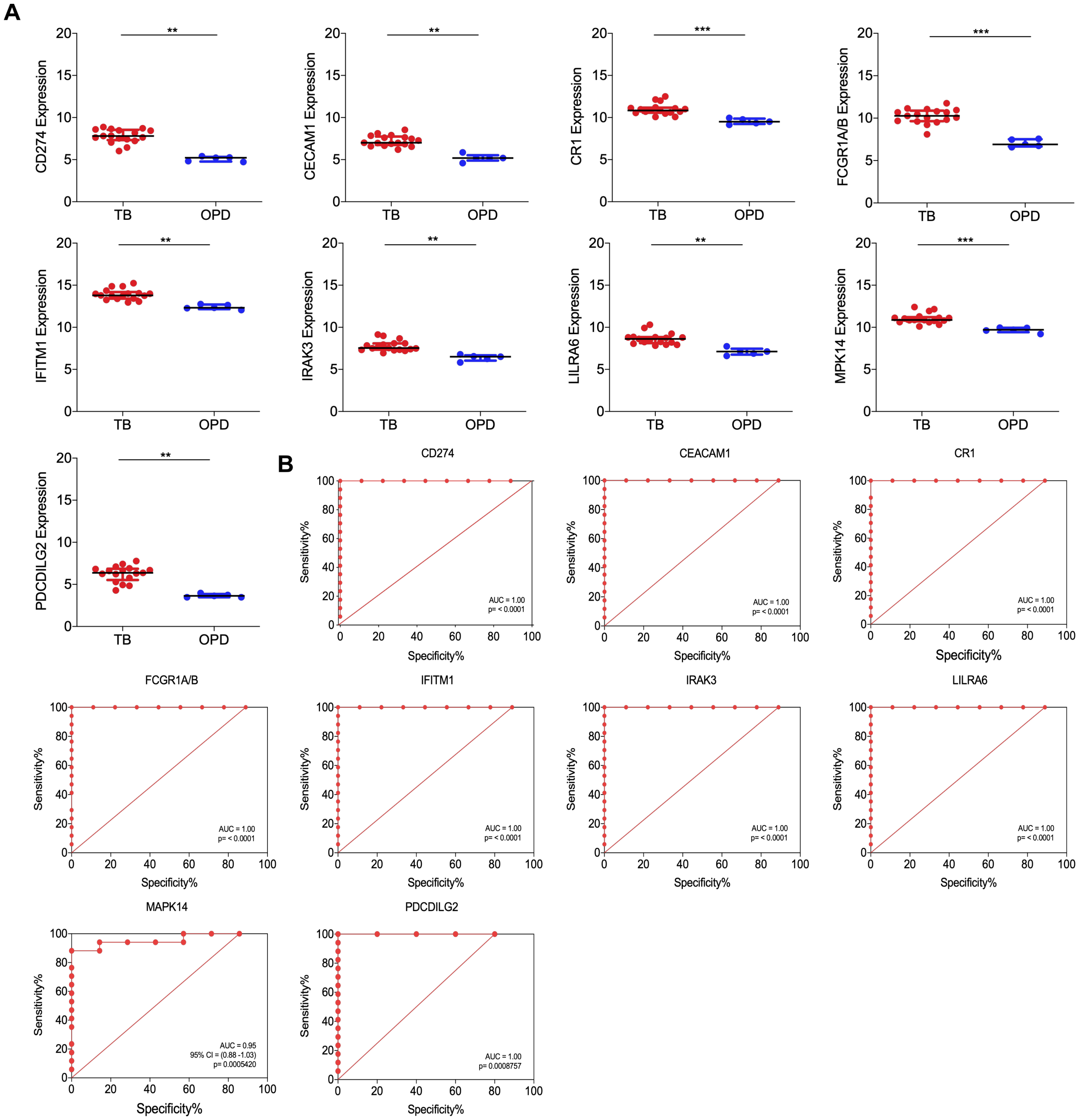

**Supplementary Figure S1. Analysis of ROC curve for TB diagnosis markers.** A. Differences in gene expression levels between TB and OPD donors. B. ROC analysis to identify discriminatory gene between TB and OPD patients. Scatter dot plots represent the gene expression for each individual donor. Analysis of ROC curve and comparisons by Mann–Whitney U test were performed using GraphPad Prism version 5.02 for windows, GraphPad software, San Diego, CA, USA, www.graphpad.com. The median and 95% confidence interval are indicated. ** indicates a p-value < 0.01; *** indicates a p-value < 0.001. AUC values (with 95% confidence interval). AUC, area under curve; CI, confidence interval; TB, tuberculosis patients; OPD, patients with other pulmonary disease than TB (asthma).

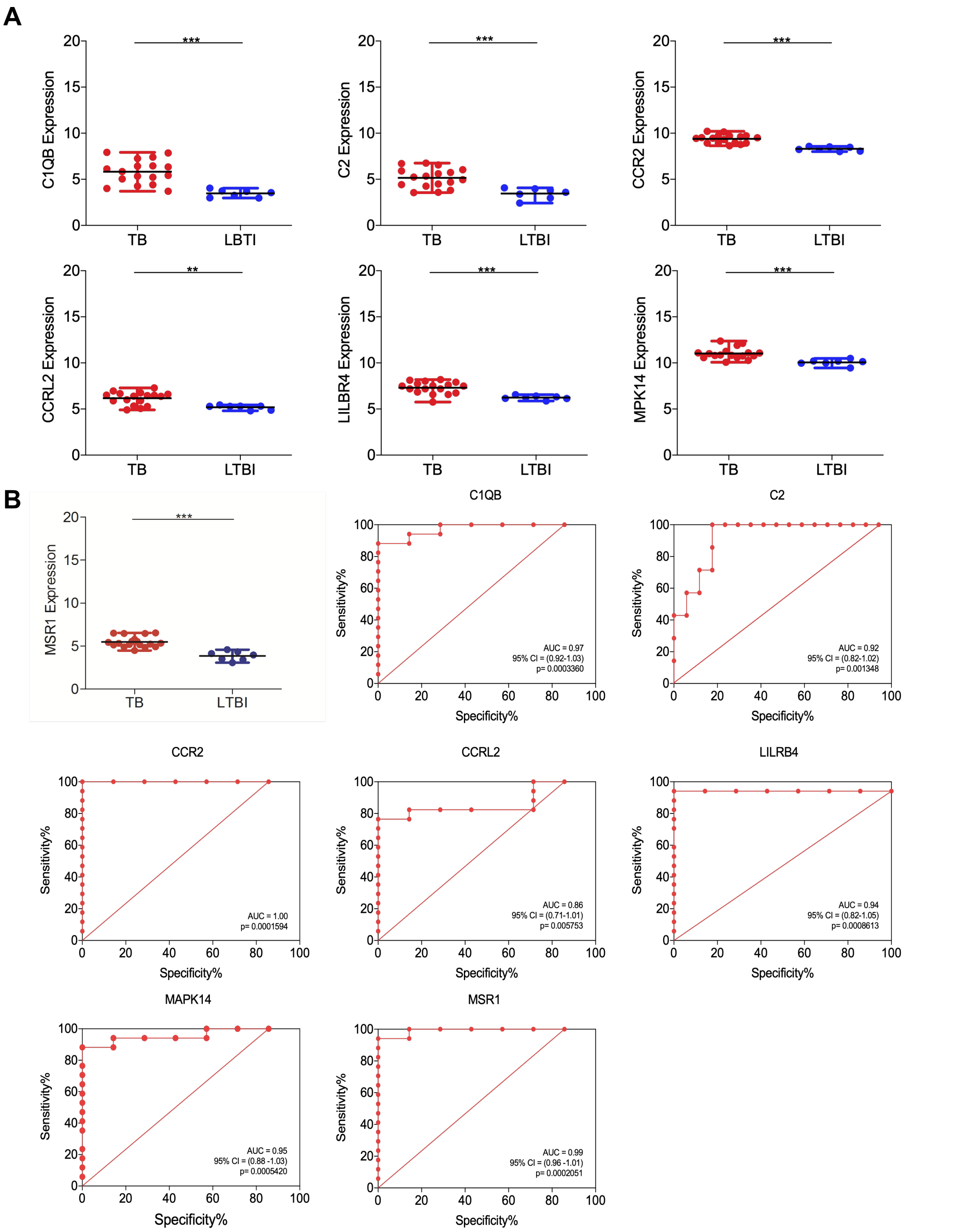


**Supplementary Figure S2. Analysis of ROC curve for TB progression markers.** A. Differences in gene expression levels between TB and LTBI donors. B. ROC analysis to identify discriminatory gene between TB and LTBI donors. Scatter dot plots represent the gene expression for each

individual donor. The median and 95% confidence interval are indicated. ** indicates a p-value < 0.01; *** indicates a p-value < 0.001. AUC values (with 95% confidence interval). AUC, area under curve; CI, confidence interval; TB, tuberculosis patients; LTBI, healthy donors latently infected with *M. tuberculosis*.
